# Structural Components for Amplification of Positive and Negative Strand VEEV Splitzicons

**DOI:** 10.3389/fmolb.2018.00071

**Published:** 2018-07-26

**Authors:** Anna K. Blakney, Paul F. McKay, Robin J. Shattock

**Affiliations:** Department of Medicine, Imperial College London, London, United Kingdom

**Keywords:** RNA, replicon, non-structural proteins, amplification, VEEV

## Abstract

RNA is a promising nucleic acid technology for both vaccines and therapeutics, and replicon RNA has gained traction as a next-generation RNA modality. Replicon RNA self-amplifies using a replicase complex derived from alphaviral non-structural proteins and yields higher protein expression than a similar dose of messenger RNA. Here, we debut RNA splitzicons; a split replicon system wherein the non-structural proteins (NSPs) and the gene of interest are encoded on separate RNA molecules, but still exhibit the self-amplification properties of replicon RNA. We designed both positive and negative strand splitzicons encoding firefly luciferase as a reporter protein to determine which structural components, including the 5′ untranslated region (UTR), a 51-nucleotide conserved sequence element (CSE) from the first nonstructural protein, the subgenomic promoter (SGP) and corresponding untranslated region, and an internal ribosomal entry site (IRES) affect amplification. When paired with a NSP construct derived from the whole, wild type replicon, both the positive and negative strand splitzicons were amplified. The combination of the 51nt CSE, subgenomic promoter and untranslated region were imperative for the positive strand splitzicon, while the negative strand was amplified simply with inclusion of the subgenomic promoter. The splitzicons were amplified by NSPs in multiple cell types and show increasing protein expression with increasing doses of NSP. Furthermore, both the positive and negative strand splitzicons continued to amplify over the course of 72 h, up to >100,000-fold. This work demonstrates a system for screening the components required for amplification from the positive and negative strand intermediates of RNA replicons and presents a new approach to RNA replicon technology.

## Introduction

RNA has emerged as a promising nucleic acid technology in the context of both vaccines and therapeutics (Kole et al., 2012; Pardi et al., 2018). Recently, replicon RNA has gained traction as the next-generation RNA modality due to the self-amplification properties, which typically yield higher protein expression for a similar dose of RNA (Geall et al., 2012; Brito et al., 2014, 2015). Alphavirus self-amplifying RNA consists of the non-structural proteins from either the Venezuelan Equine Encephalitis Virus (VEEV) (Pushko et al., 1997), Sindbis Virus (SIN) (Bredenbeek et al., 1993) or Semliki Forest Virus (SFV) (Liljeström and Garoff, 1991), the 5′ and 3′ untranslated regions (UTRs), and the native subgenomic promoter (SGP) followed by a heterologous gene of interest (GOI). When the RNA enters the cytoplasm, it is then translated to produce the replicase complex, which amplifies the original RNA template to produce many copies of the original RNA, thus enhancing the overall protein expression (Strauss and Strauss, 1994).

Upon entry of the replicon into a cell, the RNA is translated to produce four nonstructural proteins (NSPs), which combine to form the alphaviral replicase. The replicase then produces a negative strand RNA intermediate, and subsequently two distinct positive-strand RNA species. The first positive strand is a genomic-length RNA transcript, that is a direct copy of the initial full-length RNA transcript (Strauss and Strauss, 1994). The second positive strand is an abundant positive subgenomic RNA encoding the heterologous GOI. The early replication complex consisting of the NSP1-3 polyprotein and NSP4 synthesizes the negative RNA strands, while the later replication complex composed of fully processed NSP1-4 is responsible for the production of genomic and subgenomic positive strands (Kääriäinen and Ahola, 2002; Pietilä et al., 2017). The replication takes place within membrane invaginations called spherules, which concentrate the replication components and protect double-stranded RNA intermediates (Hellström et al., 2016).

Because the NSP replication complex interacts independently with the positive and negative strand RNA species, we hypothesized that it would be possible to split the replicon into two separate species; one encoding the NSPs and one encoding the heterologous GOI template. We deemed this split replicon a “splitzicon” in order to differentiate from existing replicon RNA approaches. However, it is unknown which structural components of the positive and negative strand templates are required for amplification. Hardy et al. observed that the wild-type 3′ conserved sequence element and a polyA tail with a minimum of 12 residues are required for negative strand synthesis (Hardy and Rice, 2005). Gorchakov et al. found that one or more AU repeats or short stretches of oligo(A) were more highly efficient for negative strand synthesis and replication (Gorchakov et al., 2004). Though the imperative 5′ and 3′ elements are not completely understood, there exist mechanisms to repair or restore information at the 3′ end of defective alphavirus genomes, thus these elements play a crucial role in RNA replication (Guan and Simon, 2000) and stability (Decker and Parker, 1995). The final component included in these designs is the encephalomyocarditis virus (EMCV) internal ribosomal entry site (IRES), which is not a component of the wild-type VEEV, but has been previously used to enhance expression of bicistronic genes, and functions as a control for the subgenomic UTR (SG UTR) (Bochkov and Palmenberg, 2006).

**Figure 1:**
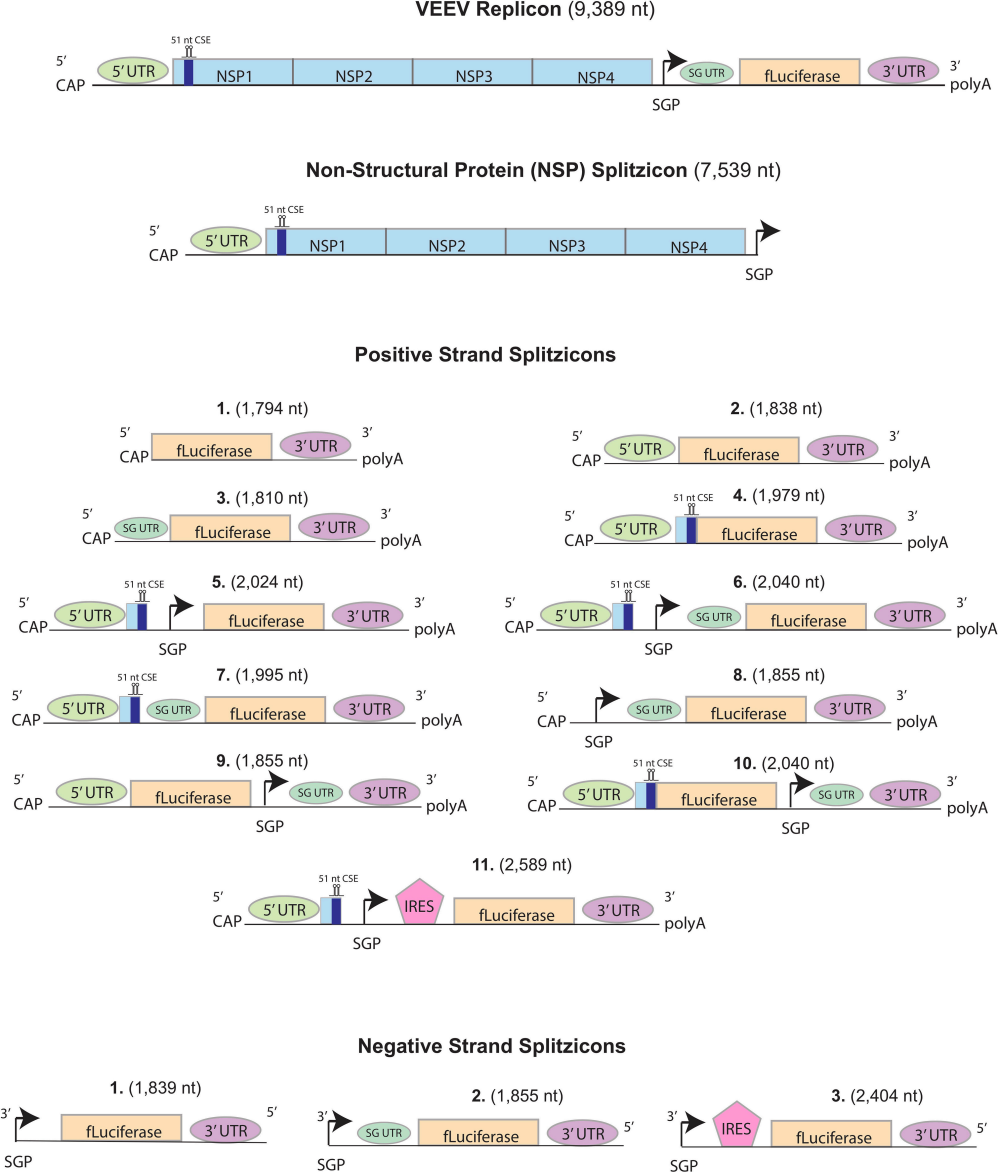
Design of positive, negative and non-structural protein (NSP) splitzicons encoding firefly luciferase. Key: CAP = m7G-cap, NSP1-4 = Non-structural proteins from the Venezuelan Equine Encephalitis Virus (VEEV), IRES = encephalomyocarditis virus internal ribosomal entry site, UTR = untranslated region, fLuciferase = firefly luciferase, black arrow = subgenomic promoter, 51nt CSE = conserved sequence element from NSP1, SG UTR = subgenomic UTR.

Here, we present RNA splitzicons that demonstrate the imperative aspects of the positive and negative strand constructs for amplification by the NSPs. For the positive strand splitzicons, we designed 11 constructs with various combinations of UTR, subgenomic promoter and corresponding UTR both before and after the GOI, the 51nt CSE from NSP1 and the EMCV IRES (Bochkov and Palmenberg, 2006; Figure 1). Because the negative strand is an intermediate RNA species, we designed three negative strand splitzicons with only the subgenomic promoter, subgenomic UTR and the EMCV IRES. We used an NSP construct that was derived directly from the wild-type VEEV replicon in order to study amplification of the splitzicons. We show the components for both the positive and negative strand splitzicons required for amplification with the NSPs, and the effects of time and increasing the NSP dose. This work presents a valuable tool for screening the components required for amplification from the positive and negative strand intermediates of RNA replicons and presents a new approach to RNA replicon technology.

## Materials and methods

### Positive and negative strand splitzicon construct synthesis

Positive and negative strand designs were synthesized by GeneArt (Invitrogen, UK) with a NdeI and MluI restriction site at either end of the construct. The correct sequence was confirmed by Sanger sequencing.

### NSP splitzicon construct synthesis

The NSP splitzicon was derived from the whole VEEV replicon, by linearizing immediately after the subgenomic promoter with NdeI.

### Plasmid DNA synthesis and purification

pDNA was transformed into *Escherichia coli* and grown in 50 mL cultures in LB with 100 μg mL^−1^ carbenicillin [Sigma Aldrich, UK, (NSP splitzicon)] or 100 μg mL^−1^ kanamycin [Sigma Aldrich, UK, (positive and negative strand splitzicons)]. pDNA was isolated and purified using a Plasmid Plus Maxiprep kit (QIAGEN, UK). pDNA concentration and purity were measured on a NanoDrop One (ThermoFisher, UK) prior to use with *in vitro* transcription reactions.

### RNA preparation and purification

pDNA was linearized using the NdeI and MluI restriction sites for 2 h at 37°C, following heat inactivation at 80°C for 20 min. Capped i*n vitro* RNA transcripts were synthesized by adding 1 μg of linearized DNA to a mMessage mMachine reaction (Promega, UK) with an additional 1 μL GTP (3 mM), according to the manufacturer's protocol, for the NSP splitzicon construct in each reaction in order to increase the yield. *In vitro* transcription reactions wherein the transcripts are capped in the same reaction are limited by GTP, and this is especially limiting for large constructs, such as the NSP splitzicon. Each reaction was incubated for 4 h at 37°C. RNA was then purified using a MEGAClear Transcription Clean-Up Kit (Thermo, UK) according to the manufacturer protocol. RNA concentration and purity were measured on a NanoDrop One prior to transfection.

### Cell culture

HEK293T.17 (ATCC, USA) or A549 (ATCC, USA) cells were cultured in complete Dulbecco's Modified Eagle's Medium (DMEM) (Gibco, ThermoFisher, UK) containing [10% fetal bovine serum, 5 mg mL^−1^ L-glutamine, 5 mg mL^−1^ penicillin streptomycin (ThermoFisher, UK)].

### *In vitro* transfections

A stock solution of PEI MAX™ (Polysciences, Germany), a linear polyethyleneimine transfection reagent with a molecular weight of 40,000 Da, was prepared at a concentration of 2 mg mL^−1^ and a pH of 7 in ultrapure H_2_O and filtered using a 0.22 μm syringe filter (Millipore, Sigma, UK). RNA complexes were prepared by diluting the polymer and RNA into equal volumes of DMEM with 0.5 mg mL^−1^ L-glutamine, adding the PEI solution to the RNA solution using a pipette, and immediately vortexing for 30 s. The ratio of PEI to total RNA, including positive/negative/NSP splitzicons, was fixed at a ratio of 20:1. HEK 293T.17 or A549 cells were plated at a density of 50,000 cells well^−1^ 48 h prior to transfection. The RNA complexes were added to each well in a total volume of 100 μL and a total dose of 100 ng of positive/negative splitzicon with or without 100 ng of NSP splitzicon, unless otherwise specified. Cells were allowed to transfect for 4 h, and then the media was replaced with 100 μL of complete DMEM [10% fetal bovine serum, 5 mg mL^−1^ L-glutamine, 5 mg mL^−1^ penicillin streptomycin (ThermoFisher, UK)] until the appropriate timepoint.

### Luciferase assay

After 4, 8, 24, or 72 h from the initial time of transfection, 50 μL of media was removed from each well and 50 μL of ONE-Glo™ luciferase substrate (Promega, UK) was added and mixed well. Then, the total 100 μL was transferred to a white 96-well plate and analyzed on an FLUOstar Omega plate reader (BMG LABTECH, UK) with a gain of 4000. The average of three control wells was subtracted from each value to account for any auto-luminescence from the cells.

### Statistical analysis

Statistical analysis was performed using Prism 7 (GraphPad), using α = 0.05 to indicate significance.

## Results

### 51nt CSE, subgenomic promoter and untranslated region are required for amplification of positive strand splitzicon

**Figure 2:**
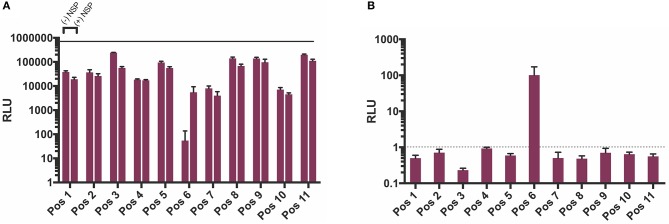
NSP-induced amplification of positive strand splitzicons in HEK cells 24 h after transfection. **(A)** Luminescence represented as mean ± standard deviation RLU with and without 0.1 ug of NSP. **(B)** Relative amplification of positive strand constructs, normalized to no NSP control. Solid line in **(A)** represents the luciferase expression by the whole replicon, while dotted line at 1in **(B)** represents no change over the no NSP control for the same condition.

**Figure 3:**
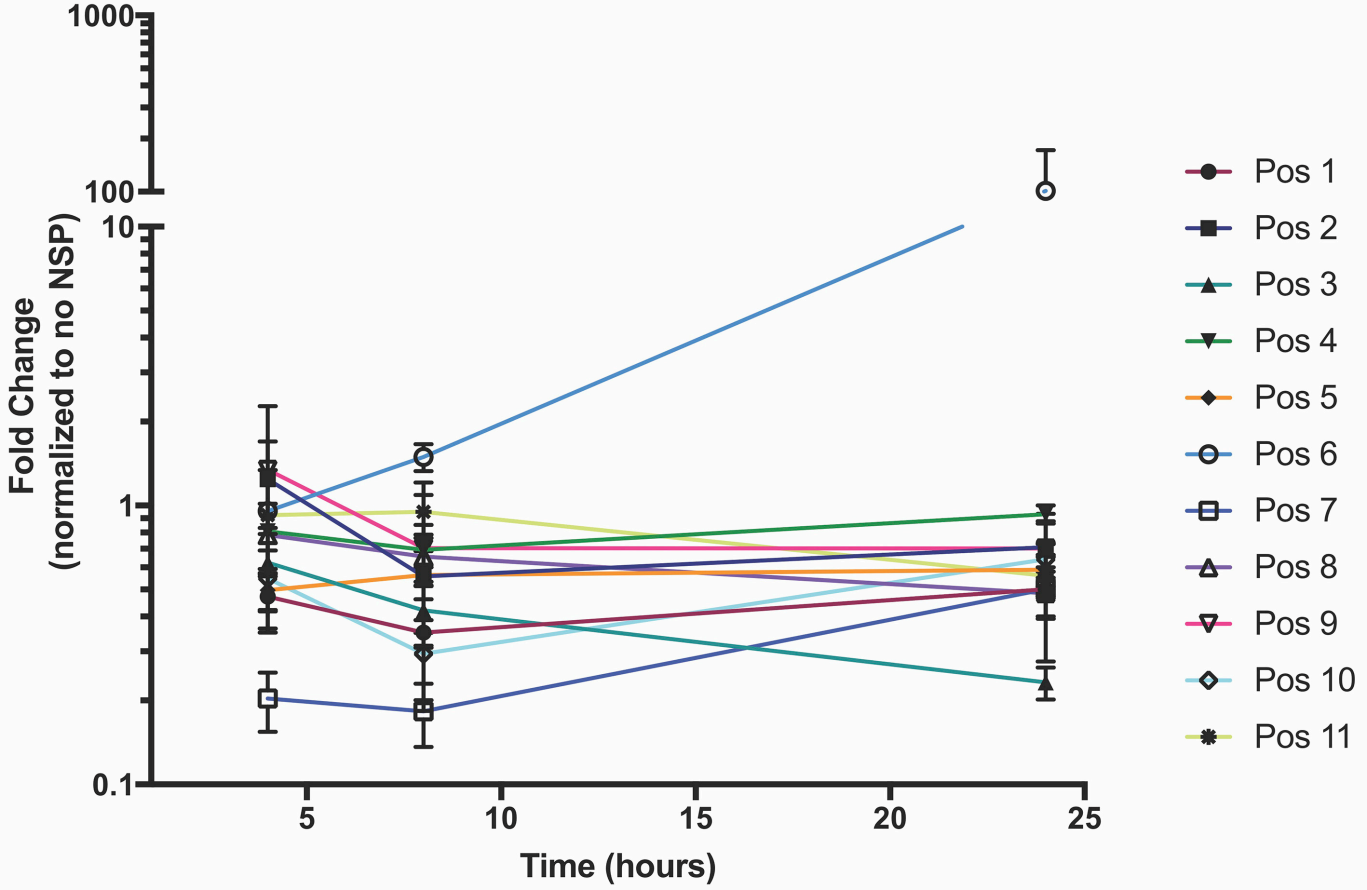
NSP-induced amplification of positive strand splitzicons in HEK cells over the course of 24 h. Values represent mean fold change ± standard deviation, and RLU values are normalized to the same condition without added NSP splitzicon.

In order to test which components of the positive strand splitzicon are imperative for amplification, we designed a series of constructs with different combinations of the untranslated region, 51 nucleotide conserved sequence element from NSP1, subgenomic promoter and corresponding untranslated region, and EMCV IRES (Figure 1 and Supplementary Table 1). We tested a dose of 100 ng of the positive strand splitzicons with and without an equivalent dose of the NSP splitzicon to observe whether the NSPs amplified the positive splitzicon, and what components were necessary for amplification (Figure 2). All of the positive strand splitzicons expressed luciferase independently of co-delivery with the NSP splitzicon (Figure 2A). However, the positive splitzicon with the 51 nt CSE, subgenomic promoter and subgenomic untranslated region (Pos 6) had 3 to 4-fold lower expression than the other constructs. A fold change greater than one when normalized to the condition without a dose of NSP indicates amplification (Figure 2B). Only Pos 6 was amplified ~100-fold compared to the no NSP condition. The rest of the positive splitzicons had a fold change equal to or less than one, indicating interference of luciferase expression from the positive strand when co-delivered with the NSP construct. Compared to the whole replicon, none of the positive strand splitzicons achieved the same amount of luciferase expression, even in the presence of the NSPs. Because of the observed lack of amplification at 24 h, we then sought to determine whether there was any amplification of the positive splitzicons at earlier timepoints (Figure 3). While Pos 2 and Pos 9 showed a small degree of amplification (1.3 and 1.5-fold, respectively) after 4 h, this trend dissipated after 8 h. While Pos 6 showed no amplification after 4 h, and slightly enhanced amplification (1.6-fold) after 8 h, this trend continued to increase to ~100-fold amplification at 24 h.

### Amplification of negative strand splitzicon is conferred by subgenomic promoter alone

**Figure 4:**
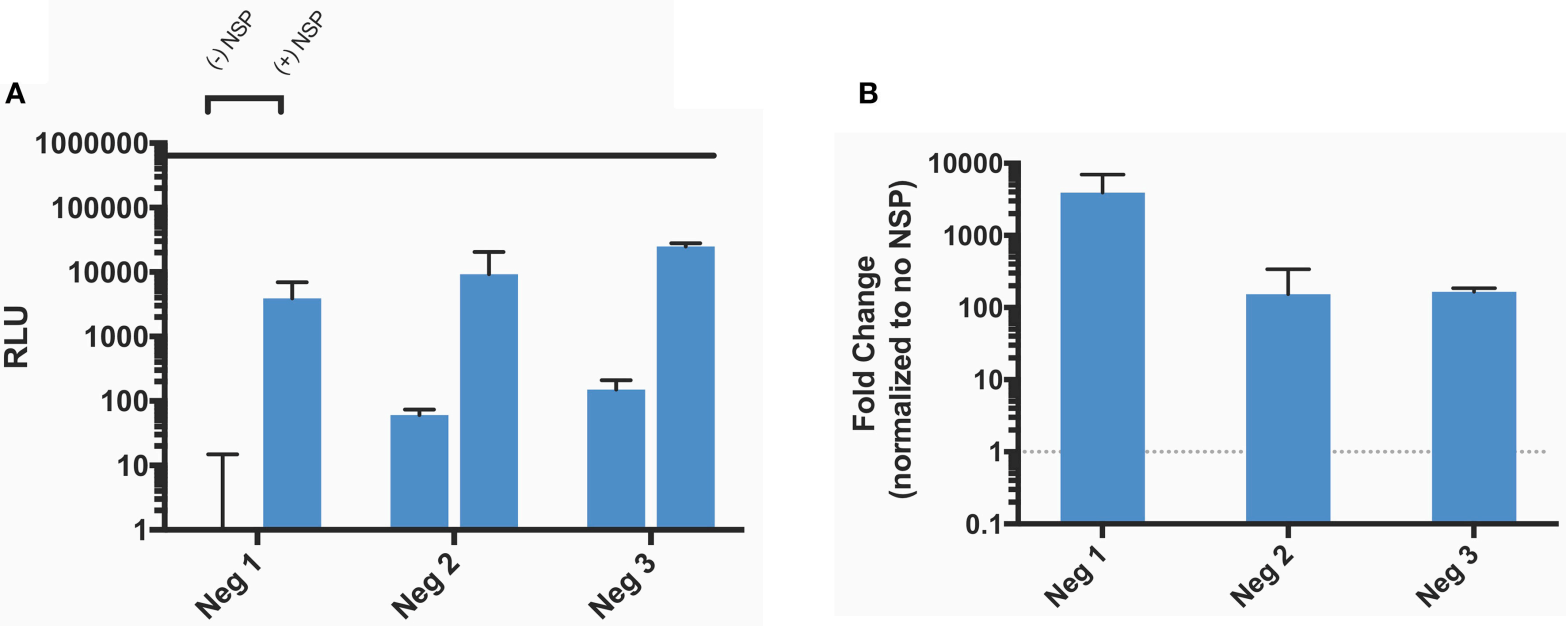
Amplification of negative strand splitzicons by NSP in HEK cells 24 h after transfection. **(A)** Luminescence represented as mean ± standard deviation RLU with and without 0.1 ug of NSP. **(B)** Relative amplification of negative strand constructs, normalized to no NSP control. Solid line in **(A)** represents the luciferase expression by the whole replicon, while dotted line at 1 represents no change over the no NSP control for the same condition.

Because the VEEV replicase complex uses the original positive strand transcript as a template to make a negative strand subgenomic copy, we also designed three negative strand splitzicons to assess the components required for negative strand amplification (Figure 1). Similarly to the positive strand splitzicons, we then administered a dose of 100 ng and assessed which components resulted in amplification when co-delivered with the NSP splitzicon at an equivalent dose (Figure 4). Unlike the positive strand splitzicons, the negative strand splitzicons hardly expressed luciferase in the absence of the NSPs (Figure 4A); all three were at or below 100 RLU. However, when combined with an equivalent dose of the NSP splitzicon, all three negative splitzicons were amplified between 100- and 1,000-fold (Figure 4B). Because the negative splitzicon with only the SGP (Neg 1) had a negligible signal, a value of 10 RLU was used with which to normalize the condition with the NSP splitzicon, as to provide a conservative estimate of the magnitude of amplification. Though Neg 1 had the highest amplification, Neg 3 had the highest overall luciferase expression. There was no added benefit of addition of the SG UTR after the SGP for the negative strand splitzicon after 24 h. Likewise to the positive strand splitzicons, none of the negative strand splitzicons had equivalent luciferase expression to the whole replicon.

### Splitzicon expression and amplification translates to other cell types

**Figure 5:**
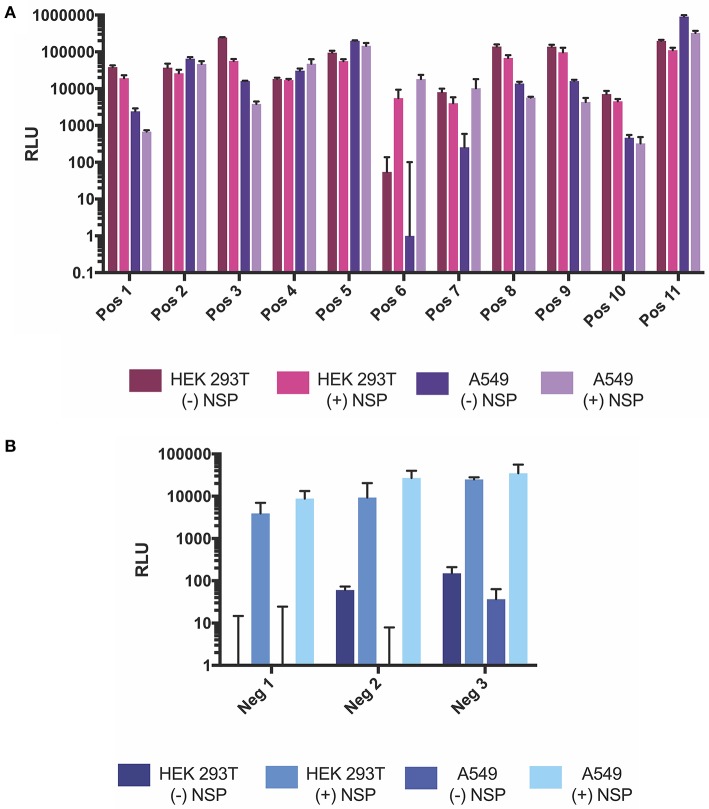
Comparison of fLuciferase expression after 24 h from positive **(A)** and negative **(B)** strand splitzicons with and without NSP in HEK 293T and A549 cells. Transfections were performed either without or with 0.1 ug NSP splitzicon, and results are represented at mean ± standard deviation RLU.

As HEK293T cells are known to be easily transfected (Thomas and Smart, 2005), we sought to test whether the positive and negative strand splitzicons were amplified in other cells as well. Additionally, the HEK293T.17 cells used in these studies are known to constitutively express the simian virus 40 (SV40) large T antigen, which could affect RNA replication (Schirrmann and Büssow, 2010). A dose of 100 ng of positive and negative strand splitzicons were transfected with and without an equivalent dose of the NSP splitzicon in A549 cells, a human adenocarcinomic alveolar basal epithelial cell line (Figure 5). The trends in A549 cells were similar to those in HEK cells for both the positive and negative strand splitzicons. All of the positive strand splitzicons expressed luciferase in the absence of the NSP splitzicon, though Pos 6 expression was lower in A549 cells than HEK cells, and only Neg 3 had expression levels above the limit of detection for the assay. When co-delivered with the NSP splitzicon, all the negative strand splitzicons were amplified to similar levels in HEK and A549 cells, while only Pos 6 was amplified. Although the values of luciferase expression were similar between the A549 and HEK cells, Pos 1, 3, 7, 8, 9, and 10 expressed an order of magnitude higher in HEK cells, while Pos 11 was an order of magnitude higher in A549 cells. These data show that the amplification of positive and negative strand splitzicons are reproducible within different cell types and not just an artifact of promiscuous HEK cells, but there are cell specific expression effects between the splitzicons.

### Negative strand splitzicon exhibits linear, dose dependent expression with increasing NSP splitzicon dose

**Figure 6:**
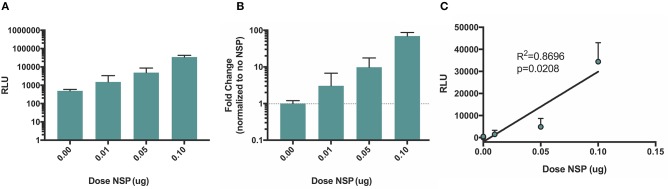
Amplification of negative strand splitzicon increases linearly with increasing doses of NSP. Negative strand was dosed at a 0.05 ug with increasing doses of NSP; **(A)** expressed as mean ± standard deviation RLU, **(B)** fold change compared to the control with no NSP and **(C)** linear fit of luciferase expression versus NSP dose. Dotted line at 1 represents no change over the no NSP control for the same condition.

Although the amplification of both positive and negative strand splitzicons is evident by comparing the luciferase expression with and without the NSP splitzicon, we sought to determine whether increasing the dose of the NSP splitzicon increased the amplification. We co-delivered a dose of 50 ng of the negative strand splitzicon (Neg 1) with doses of the NSP splitzicon from 10 to 100 ng and observed the luciferase expression after 24 h (Figure 6). Both the luciferase expression (Figure 6A) and fold-change compared to the condition without the NSP splitzicon (Figure 6B) increased with increasing dose of the NSP splitzicon. These data confirm that the NSP splitzicon is the limiting factor in amplification and resulting luciferase expression. The luciferase expression data was fit with a linear regression to the NSP dose, to assess the linear relationship between the amount of NSP splitzicon and amplification (Figure 6C). We observed a significant positive linear correlation (*p* = 0.0208) with an *R*^2^-value of 0.8696. This linear relationship indicates that a lower dose of the negative strand splitzicon could potentially be used to yield the same luciferase expression, and that the equivalent doses of negative and NSP splitzicons, as delivered when using the whole replicon, may not be the optimal ratio.

### Amplification of positive and negative strand splitzicons continually increases over 72 h

**Figure 7:**
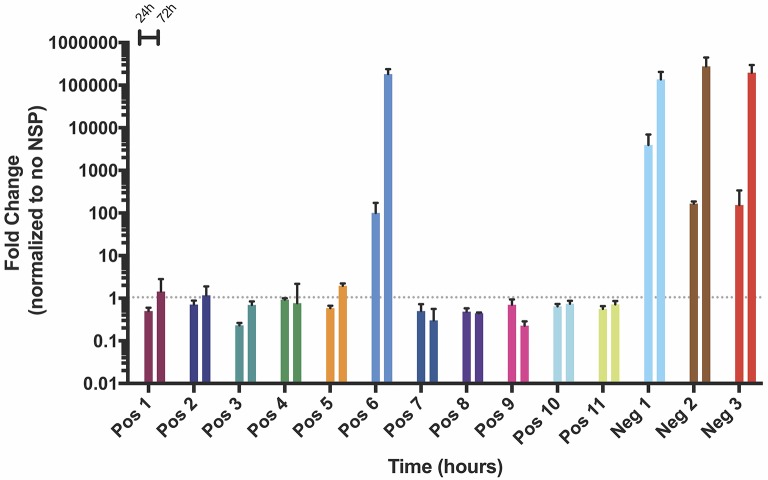
Change in NSP amplification of positive and negative strand constructs in A549 cells over the course of 3 days, expressed as mean ± standard deviation normalized to the no NSP control at the same timepoint. Gray dotted line indicates no change (fold change = 1).

After observing amplification of both positive and negative strand splitzicons over 24 h, we aimed to characterize whether this trend was sustained over the course of 72 h. Because HEK cells begin to lift off the plate due to over-confluence after 72 h, A549 cells were transfected with a dose of 100 ng of all of positive and negative strand splitzicons (Figure 1) and an equivalent dose of the NSP splitzicon (Figure 7). While Neg 1 exhibited 2,500-fold amplification after 24 h, Pos 6, Neg 2, and Neg 3 all exhibited ~100-fold amplification. After 72 h, the Pos 6 and all three negative splitzicons had equivalent amplification of 100,000-fold over the condition with no NSP splitzicons (Supplementary Figure 1). These data suggest that a positive or negative splitzicon with the correct components and a certain dose of NSP splitzicon can amplify the RNA up to a certain threshold over 72 h *in vitro*.

## Discussion

Here we demonstrate that the splitzicon system, consisting of separate NSP and heterologous gene RNA species, is capable of RNA amplification and higher protein expression than the mRNA alone. These data indicate which components of the splitzicons are required for amplification. For the positive strand splitzicon, the combination of the 51nt CSE, SGP and SG UTR resulted in amplification, while the EMCV IRES and combinations of SGP, SG UTR and 51nt CSE in duality did not. On the contrary, all three negative strand splitzicon designs were amplified by the NSP, thus indicating that SGP alone allowed amplification, which was not enhanced by the SG UTR nor the EMCV IRES. We demonstrate that splitzicon amplification is observed in two cell types, indicating that the system is robust and translatable. The amplification of the negative strand splitzicon was found to increase linearly with increasing dose of NSPs, implying that the NSPs are the limiting factor in the amplification. Furthermore, the amplification increases over the course of 3 days for both the positive and negative strand splitzicons. In total, these data validate the splitzicon system and demonstrate that both the positive and negative strand splitzicons are amplified by NSPs.

As expected, the positive strand splitzicons results in luciferase expression in the absence of NSPs, while the negative strand splitzicons did not. However, the expression of Pos 6 was hampered compared to the other positive strand constructs (Figure 2). This difference in positive splitzicon protein expression was rescued by addition of the NSPs. The observation that inclusion of the SG-UTR (Pos 6) leads to low luciferase expression in the absence of NSP amplification, suggests that it acts as a negative regulator of SG-mRNA translation. Thus, the structure of the SG-UTR after the SG-promoter initiation sequence seems to hamper protein synthesis (Supplementary Figure 2). However, this appears to be context specific as there is no reduction in expression when the SG-promotor is omitted (Pos 7) or when the SG-promotor and SG-UTR are configured independently of any upstream sequence (Pos 8). Here the lack of any upstream sequence may facilitate melting of the secondary structure of the SG-UTR allowing translation to proceed unhampered. This is also consistent with efficient translation of SG-mRNA following its amplification by the NSPs, whether from positive (Pos 6) or negative templates (Neg 2). This is reminiscent of the regulation of the translation of Sindibis SG-mRNA regulated by a hairpin structure before, rather than after the SG initiation codon (Garcia-Moreno et al., 2015). This novel, context specific, interaction between the SG-promoter and SG-UTR in the regulation of SG-mRNA merits further investigation. We postulate that the secondary structure in Pos 6 conferred amplification as this is the native order of these components in the wild type VEEV: 5′ UTR followed by a 51 nt CSE in NSP1, the subgenomic promoter and corresponding UTR. The amplification of the positive strand splitzicon is similar to that observed by Spuul et al. using a DNA-launched SFV replicase complex and positive strand template (Spuul et al., 2011). Although the 51 nt CSE is thought to enhance both positive and negative strand synthesis (Frolov et al., 2001; Nickens and Hardy, 2008), it did not confer amplification alone or any appreciable benefit to expression from the positive strand splitzicons. The subgenomic promoter is a known conserved sequence element within the alphavirus family (Rupp et al., 2015), and was found to impact both positive and negative strand amplification.

The negative strand splitzicons were all amplified by NSP addition. It is unclear whether the NSPs amplify the negative strand splitzicons more abundantly because this process does not require an RNA intermediate, whereas the positive strand splitzicons would have to undergo conversion to a negative strand, which is susceptible to degradation if not properly located in spherules (Kallio et al., 2013). Hellström et al. observed that the negative strand was not able to serve as a template for the replicase complex, however, the negative strand designs used in that study were simply direct reversals of the positive strand template and did not have the SGP in the orientation that we found to be imperative (Hellström et al., 2016).

We observed that the amplification of negative strand splitzicons was enhanced by increasing the dose of NSPs. This indicates that the amplification is limited by the amount of the NSPs present in the cell and suggests that providing an abundance of NSPs could further enhance amplification and protein expression from a given dose of positive or negative strand splitzicons. Furthermore, we observed that a higher dose of Neg 1 (100 ng, Figure 4) resulted in higher amplification than a lower dose of Neg 1 (50 ng, Figure 6), when delivered with the same ratio of NSP splitzicon to negative strand template. This indicates that the total luciferase expression is also impacted by the initial splitzicon template available. Other studies have suggested that the NSPs are abundant relative to the genomic and subgenomic copies during alphaviral replication (Jose et al., 2009); however this evidence suggested that this relationship differs in the splitzicon system compared to viral replication. It is possible that this could be due to the difficulty of intracellular RNA delivery, which is especially limited for large RNA transcripts such as the NSP splitzicon. Additionally, alphavirus replication and production of the negative strand is isolated in spherules wherein the apparent concentration of the replicase may be considerably greater than in the splitzicon system (Frolova et al., 2010). While there have been extensive studies on the function of each of the four NSPs (Kääriäinen and Ahola, 2002; Rupp et al., 2015), the ratio between the NSPs and template is usually fixed when delivered as a single replicon molecule and the optimal ratio between the replicase and template has not been defined. After 3 days, both the positive and negative strand splitzicons were amplified by ~100,000-fold (Figure 7), which potentially shows that there is a maximum amplification capacity for the NSPs *in vitro*.

While there have been previous reports of DNA launched replicase/template systems (Lemm and Rice, 1993; Lemm et al., 1998; Spuul et al., 2011; Hellström et al., 2016), this is the first report of a splitzicon system delivered directly to cells in the form of RNA. This system could potentially be used for a number of applications, including further characterization of the components on the positive and negative strand templates that confer amplification, which could be applied to either splitzicons or the whole replicon, or expression of multiple genes from templates that encode multiple heterologous genes as an IRES alternative (Bochkov and Palmenberg, 2006). Encoding multiple genes with splitzicon templates could be useful tools in the context of RNA vaccines for multivalent antigen delivery or combination of antigen and adjuvant components. These data warrant further research on the co-localization of positive/negative and NSP splitzicons within cells and the optimal delivery platforms for these constructs.

## Author contributions

AB, PM, and RS conceived of and designed the experiments. AB performed the experiments and analyzed the data. AB, PM, and RS wrote the paper.

### Conflict of interest statement

The authors declare that the research was conducted in the absence of any commercial or financial relationships that could be construed as a potential conflict of interest.
