# Supplementary material for: Structural Components for Amplification of Positive and Negative Strand VEEV Splitzicons
Source: Front Mol Biosci. 2018 Jul 26;5:71. doi: 10.3389/fmolb.2018.00071 (PMC6070733; doi:10.3389/fmolb.2018.00071)
Supplement: Supplementary file 1 [file Table_1.DOCX]

Supplementary Material

Structural components for amplification of positive and negative strand VEEV splitzicons

Anna K. Blakney^1^, Paul F. McKay^1^, Robin J. Shattock^1,*^

*** Correspondence:** Robin J. Shattock, r.shattock@imperial.ac.uk

# Supplementary Figures

**
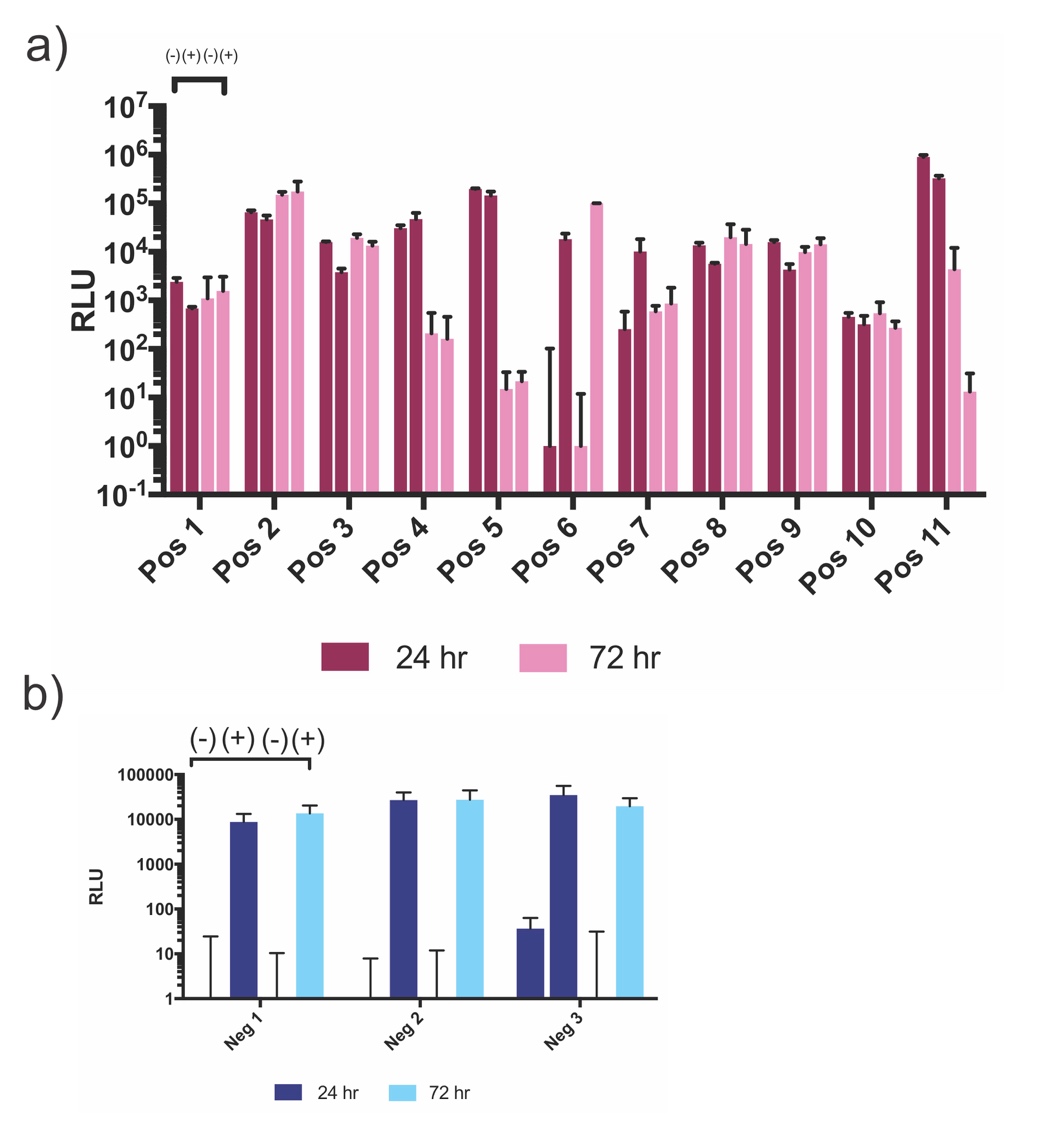
**

**Supplementary Figure 1.** Comparison of fLuciferase expression after 24 and 72 hours from positive (a) and negative (b) strand splitzicons with and without NSP in A549 cells. Transfections were performed either without or with 0.1 ug NSP splitzicon and results are represented as mean ± standard deviation RLU.


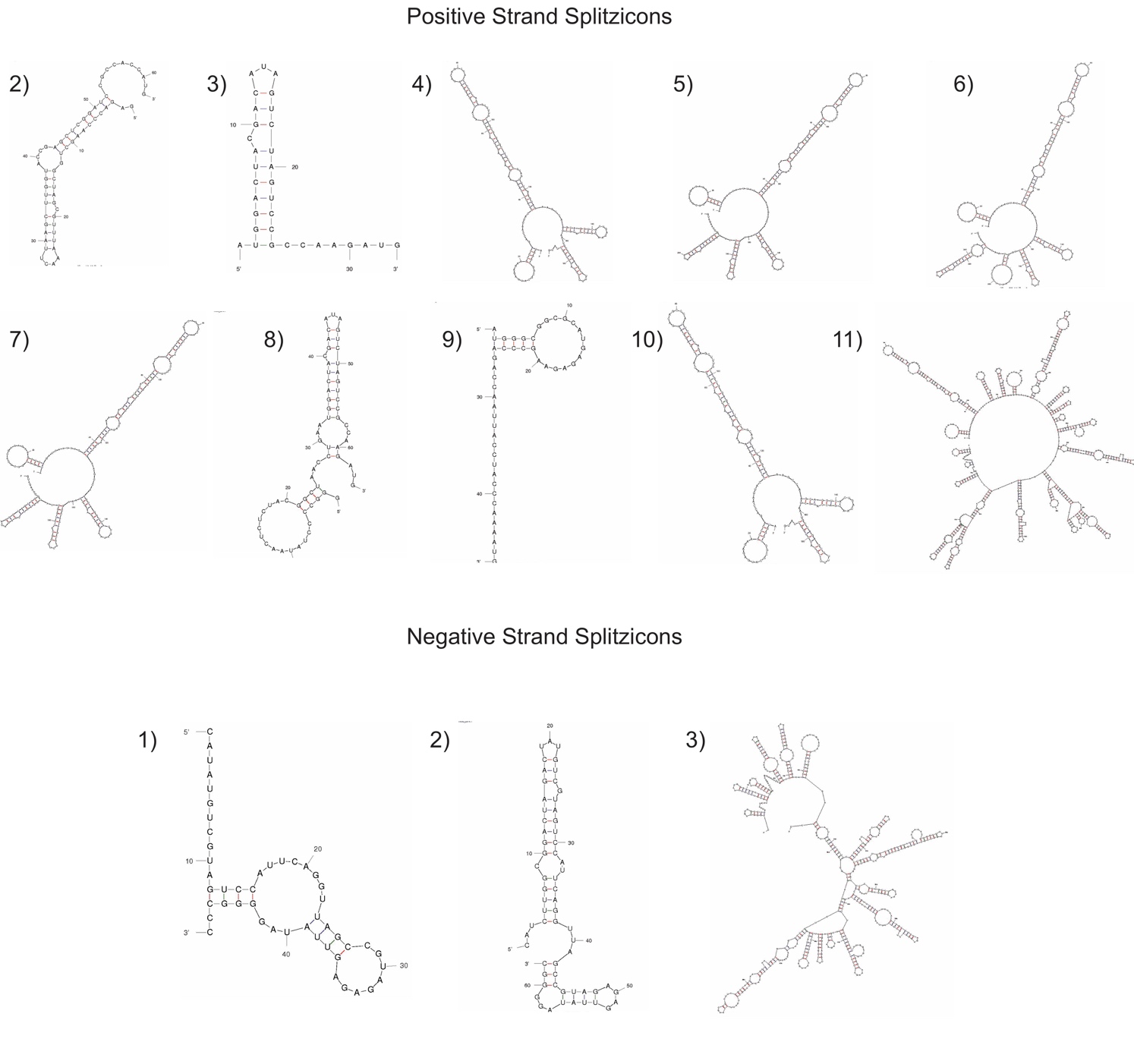


**Supplementary Figure 2.** Secondary structure region upstream of fLuciferase gene of interest of positive and negative strand splitzicons, as determined by mFold software.

**Supplementary Table 1.** Sequences of structural elements used in positive and negative strand splitzicons.

| **Element** | **Sequence** |
| --- | --- |
| 5’ UTR | ATGGGCGGCGCATGAGAGAAGCCCAGACCAATTACCTACCCAAA |
| 51nt CSE | AAGGAGAAAGTTCACGTTGACATCGAGGAAGACAGCCCATTCCTCAGAGCTTTGCAGCGGAGCTTCCCGCAGTTTGAGGTAGAAGCCAAGCAGGTCACTGATAATGACCATGCTAATGCCAGAGCGTTTTCGCATCTGGCT |
| SGP | GGGCCCCTATAACTCTCTACGGCTAACCTGAATGGACTACGACAT |
| SG UTR | AGTCTAGTCCGCCAAG |
| 3’ UTR | GCGGCCGCGAATTGGCAAGCTGCTTACATAGAACTCGCGGCGATTGGCATGCCGCCTTAAAATTTTTATTTTATTTTTCTTTTCTTTTCCGAATCGGATTTTGTTTTTAATATTTC |
| IRES | TAACGTTACTGGCCGAAGCCGCTTGGAATAAGGCCGGTGTGCGTTTGTCTATATGTTATTTTCCACCATATTGCCGTCTTTTGGCAATGTGAGGGCCCGGAAACCTGGCCCTGTCTTCTTGACGAGCATTCCTAGGGGTCTTTCCCCTCTCGCCAAAGGAATGCAAGGTCTGTTGAATGTCGTGAAGGAAGCAGTTCCTCTGGAAGCTTCTTGAAGACAAACAACGTCTGTAGCGACCCTTTGCAGGCAGCGGAACCCCCCACCTGGCGACAGGTGCCTCTGCGGCCAAAAGCCACGTGTATAAGATACACCTGCAAAGGCGGCACAACCCCAGTGCCACGTTGTGAGTTGGATAGTTGTGGAAAGAGTCAAATGGCTCTCCTCAAGCGTATTCAACAAGGGGCTGAAGGATGCCCAGAAGGTACCCCATTGTATGGGATCTGATCTGGGGCCTCGGTGCACATGCTTTACATGTGTTTAGTCGAGGTTAAAAAACGTCTAGGCCCCCCGAACCACGGGGACGTGGTTTTCCTTTGAAAAACACGATGATAATATGGCCACAACC |
